# A Game Theory-Based Approach for Modeling Autonomous Vehicle Behavior in Congested, Urban Lane-Changing Scenarios

**DOI:** 10.3390/s21041523

**Published:** 2021-02-22

**Authors:** Nikita Smirnov, Yuzhou Liu, Aso Validi, Walter Morales-Alvarez, Cristina Olaverri-Monreal

**Affiliations:** 1 Chair ITS-Sustainable Transport Logistics 4.0, Johannes Kepler University, 4040 Linz, Austria; yuzhou.liu@jku.at (Y.L.); aso.validi@jku.at (A.V.); walter.morales_alvarez@jku.at (W.M.-A.); 2 Department of Communications Technology, Ural Federal University, 620078 Yekaterinburg, Russia

**Keywords:** game theory, lane change, traffic jam, intelligent transport systems

## Abstract

Autonomous vehicles are expected to display human-like behavior, at least to the extent that their decisions can be intuitively understood by other road users. If this is not the case, the coexistence of manual and autonomous vehicles in a mixed environment might affect road user interactions negatively and might jeopardize road safety. To this end, it is highly important to design algorithms that are capable of analyzing human decision-making processes and of reproducing them. In this context, lane-change maneuvers have been studied extensively. However, not all potential scenarios have been considered, since most works have focused on highway rather than urban scenarios. We contribute to the field of research by investigating a particular urban traffic scenario in which an autonomous vehicle needs to determine the level of cooperation of the vehicles in the adjacent lane in order to proceed with a lane change. To this end, we present a game theory-based decision-making model for lane changing in congested urban intersections. The model takes as input driving-related parameters related to vehicles in the intersection before they come to a complete stop. We validated the model by relying on the Co-AutoSim simulator. We compared the prediction model outcomes with actual participant decisions, i.e., whether they allowed the autonomous vehicle to drive in front of them. The results are promising, with the prediction accuracy being 100% in all of the cases in which the participants allowed the lane change and 83.3% in the other cases. The false predictions were due to delays in resuming driving after the traffic light turned green.

## 1. Introduction

At this point in time, numerous studies have proven the effectiveness of autonomous vehicles in dealing with challenges such as road safety, fuel consumption, sustainability, etc. [1,2]. Considering the scientific achievements so far, it is difficult to imagine a future where autonomous vehicles will not be used in transportation. However, before introducing self-driving cars on real roads on a large scale, many problems need to be addressed and solved [3].

In the near future, we can expect autonomous and manually driven cars to coexist on most roads. Drivers operating conventional vehicles might expect human-like behavior from autonomous vehicles, which could cause situations of uncertainty and mistrust [4] if the expectations are not fulfilled, and this could ultimately threaten road safety [5]. This is particularly important in certain complex scenarios such as lane-changing maneuvers, in which cooperation with other road users is required. To address this issue, it is vital to design algorithms that are able to analyze human decision-making processes and interaction patterns as well as able to implement models to predict the action of drivers. Such algorithms can be designed by relying on game theory [6] or mathematical models to analyze interactions among several players that make decisions.

A brief description of the game theory is presented in the following paragraph. Game theory is frequently used as a core decision-making layer of autonomous vehicle algorithms [3,7,8]. The participants in the game compete with each other to protect their interests by utilizing a strategy based on the current outcome of the game and their knowledge. For lane-changing scenarios, dynamic noncooperative games are most commonly used. The game itself is a mathematics object that is strictly defined by the following:

- a fixed number of players N={1,2,3⋯n};
- a set of strategies $S_{1},S_{2},S_{3}\cdots S_{n}$ with respective to the number of players;
- specific outcomes or payoffs for any possible combination of the strategies $\left(s_{1},s_{2},s_{3}\cdots s_{n}\right)\in S_{1}*S_{2}*S_{3}*\cdots S_{n}$ (where $s_{i}$ is a current strategy of player *i*).

The payoff function is defined on a set of all outcomes of the game $U_{i}:S_{1}*S_{2}*S_{3}*\cdots S_{n}\to R$. Any strategy $s_{i}^{*}\in S_{i}$ is optimal if $\forall s_{i}\in S_{i}$, where $s_{i}\neq s_{i}^{*}$ and $\forall (s_{1},s_{2},..s_{i-1},s_{i+1}..s_{n})$ satisfy $U_{i}\left(s_{1},\cdots s_{i-1},s_{i}^{*},s_{i+1}\cdots s_{n}\right)\geq U_{i}\left(s_{1},\cdots s_{i-1},s_{i},s_{i+1}\cdots s_{n}\right)$.

In this work, we propose a decision-making model based on a dynamic noncooperative game to investigate lane changing in the urban scenario of a congested intersection, which is achieved by the implementation of a model that predicts the decision-making process of drivers. To this end, we assume that the game’s result can be predicted if each vehicle maximizes the payoff of the interaction. The proposed scenario consists of a two-lane road with traffic at an intersection regulated by traffic lights. The left lane is designated for driving through the intersection as well as left turns. The right lane is designated only for driving straight through the intersection. In the proposed scenario the traffic flow in the left lane is much slower than in the right lane, since vehicles in the left lane need to decelerate when a vehicle turns left. The vehicles in our scenario are described as follows:

- an autonomous vehicle (EGO) (in white) that performs the lane-change maneuver and is controlled by the game theory-based strategy S;
- the following vehicle (FV) in green; and
- the leading vehicle in front of the FV, represented by LEAD (in red).

**Figure 1:**
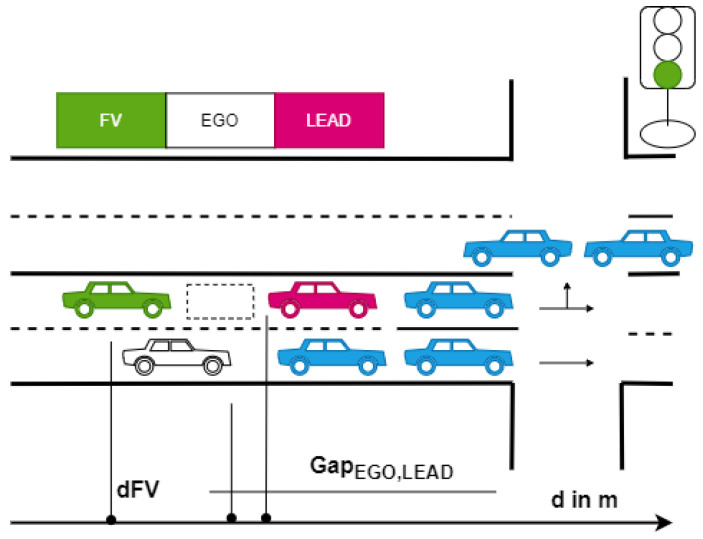
Illustration of the proposed lane-change scenario and the relevant players. The autonomous vehicle (EGO) (in white) performs a lane change to occupy the position in front of the following vehicle (FV) in green. The leading vehicle (LEAD, in red) is located in front of the FV.

We implement the following use case, as illustrated in Figure 1, which consists of the following series of actions:

1. When the traffic light is red, EGO enters the road from the right lane and stops behind the last vehicle in the queue.
2. EGO needs to perform a lane change to turn left at the intersection. Due to the traffic stopped in the left lane, EGO interacts with FV by activating the turn signal to indicate its intention to merge in front.
3. When the traffic light turns green, FV decides whether to wait and allow EGO to merge in front. This FV decision is made based on the perceived information related to the current EGO acceleration and previously conveyed information by the EGO vehicle regarding its merging intention.

According to related literature, lane-change algorithms are usually divided into the following 3 layers [9]:

- strategic layer, related to route planning;
- tactical layer, related to decision-making processes; and
- operational layer, linked to control tasks.

The proposed model focuses on the decision-making process in the tactical layer. This paper is organized as follows. In Section 2, relevant literature to lane changing in both urban and highway scenarios is reviewed and reported. In Section 3, we introduce the proposed decision-making model in detail, including the mathematical definition of payoff functions. In Section 4, the simulation environment and scenario for model validation are described. The game solution and the results obtained from the validation tests are presented in Section 5. A conclusion and future directions of the presented research are provided in Section 6.

## 2. Related Work

Decision making in autonomous vehicles (AVs) has been addressed in several works; for example, the authors in [10] developed an approach to mimic human behavior. A variety of driving styles were considered to study driving safety, ride comfort, and travel efficiency as utility functions. Two noncooperative games were implemented using Nash and Stackelberg equilibrium and later evaluated. The authors concluded that the developed algorithms were capable of performing the proper decisions under different driving situations. In [11], the authors combined game theory with reverse reinforcement learning to create an algorithm for predicting driver behavior. To this end, they relied on dynamic noncooperative game concepts and the idea of social value orientation for game formulation. The Next Generation Simulation (NGSIM) data set for highway 101 was then used to obtain the reward function.

A work based on differential games for a fully automated lane-changing and car-following control system (LCCS) was presented in [12]. The controlled vehicles made decisions in order to minimize the predicted costs that resulted from undesirable situations. The authors evaluated the discrete and continuous control variables, such as lane-change decisions and accelerations in a simulated scenario and concluded that the approach delivered optimal lane-change decisions and accelerations for both noncooperative and cooperative controllers.

A further example for lane-changing decision-making based on game theory can be found in [13]. The authors built a two-player nonzero-sum noncooperative game model under incomplete information for mandatory lane changing. Based on Harsanyi transformation [14], they transformed the model into a game that contained imperfect information in order to cover both traditional and connected environments. In order to validate the models, the authors used the NGSIM, I-80-F data set. Their model’s accuracy was 88% and 82% for their three- and two-strategy merging event models, respectively, and 77% and 61% for their three- and two-strategy models in non-merging events.

A similar approach that relied on Harsanyi transformation and the NGSIM data set described a dynamic noncooperative game [15]. Two game models for connected and for traditional environments were implemented utilizing complete and incomplete information, respectively. The game with incomplete information was then transformed to a game of imperfect information. In order to build the payoff functions, the authors used acceleration as a set of players’ strategies. In line with this, a further dynamic noncooperative game approach was implemented in [16] using acceleration as a set of strategies. Parameters such as safety and space between vehicles were in this case used to build payoff functions. The authors also incorporated driver’s aggressiveness into their model as a key factor.

As described in previous work, lane changing has been addressed in a number of studies, with the majority of them focused on highway lane-changing scenarios.

One of the first lane-change models that focused on decision-making in an urban scenario was designed by Gipps [17]. This model suggested a connected structure of decisions for the driver to follow when making a decision for lane changing. It was designed for urban traffic situations and therefore considered the influence of traffic lights, queues, and heavy duty vehicles. Although this rule-based approach was developed in the 1980s, it is still an efficient and applicable decision-making model.

Additional studies for urban scenarios were presented in [18,19]. Lane changing was studied by adopting fundamental triangular diagrams to obtain the optimal longitudinal position for autonomous vehicles in an urban intersection. The authors assumed that a lane change could not be performed when the destination lane was occupied. In real-life scenarios, however, we argue that interaction and cooperation with drivers in the destination lane might enable the maneuver. Therefore, we address exactly this situation and propose accordingly a decision-making model for a lane change in an urban intersection with dense traffic. To this end, we adhered to game theory and adopted a similar interaction process for our decision-making algorithm to the one defined in [20], in which a communication scheme with request and response messages was presented. In our work, we considered this interaction to be a competition between two drivers for a place in the traffic queue, where players make decisions based on space and safety criteria.

As previously mentioned, most of the lane-change studies performed so far are based on highway lane-changing scenarios, where in all vehicles, the lower speed limit is considered to be higher than 30 km/h (depending on the country). Consequently, the reviewed models cannot be directly applicable in more specific scenarios such as congested urban intersections.

We contribute to the research in this main field by filling in the gap in the literature regarding mandatory lane change in intersections with dense traffic in urban scenarios. To this end, we study if, contrary to what the authors in [19] assumed, a lane change can still be performed when the destination lane is occupied. We argue that, in real-life scenarios, interaction and cooperation with surrounding traffic might enable the maneuver, and we propose accordingly a decision-making model based on game theory for lane change. To this end, we adapted the model presented in [16] to implement a dynamic noncooperative game that used acceleration as part of the player set of strategies. We redesigned the payoff functions and pertinent speed and acceleration parameters for a lane-change urban scenario and produced a decision-making model based on two-player nonzero-sum noncooperative dynamic game for lane changing.

A detailed description of the proposed decision-making model is presented in the next section.

## 3. The Proposed Decision-Making Model

The authors in [21] categorized several game-theory-based models into 5 groups, namely the empirical game-theory, classic Nash equilibrium, incomplete information game, sequential game, and evolutionary game models. Based on the given classification, our proposed decision-making model contributes to the growth of the sequential group of game-theory models.

In our work, we assume that autonomous vehicles can request and obtain information from nearby cars such as current position, speed, and acceleration during the red phase of a traffic light in real time. This can be accomplished by establishing a communication from vehicle to vehicle (V2V) and from vehicle to everything (V2X), or by obtaining data from sensors such as depth cameras, lidar, sonar, etc. [9]. It is important to mention that, for the sake of simplicity, the delays related to obtaining information via sensors or communication are not considered in this work. In this section, we focus on game formulation through a mathematical definition of its payoff functions and the outcome.

### 3.1. Game Formulation

We propose a decision-making model relying on a dynamic noncooperative game model, as presented in [16]. We aim to investigate lane changing in an urban scenario with dense traffic approaching an intersection. We assume that the game’s result can be predicted if each vehicle maximizes the payoff of the interaction.

To illustrate our approach, we adhere to the scenario introduced in Section 1 (Figure 1). The strategies that can be selected by the EGO and FV vehicles are limited to two, as described in the following lines: The strategy set available for EGO includes

1. to change lanes, namely strategy A1, and
2. not to change lanes, strategy A2.

The FV’s strategy set consists of the following options:

1. accepting EGO’s lane change or strategy B1
2. rejecting EGO’s lane change or strategy B2

The EGO outcome and selected strategy are defined as follows:

- outcome $P_{xy}$
- chosen strategy *y*

The FV outcome and selected strategy are defined as follows:

- outcome $Q_{xy}$
- chosen strategy *x*

**Figure 2:**
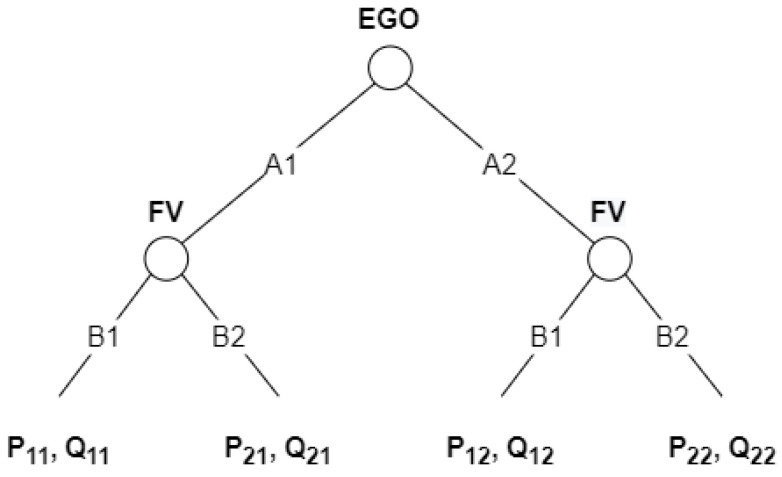
Dynamic noncooperative game description in its extensive-form representation. EGO and FV denote the players; A1 and A2, and B1 and B2 denote their respective sets of strategies. Pxy and Qxy denote the outcome of the game, where x is the FV’s selected strategy and y is the strategy selected by EGO.

The description of the game is represented in its game theory normal-form and extensive-form in Table 1 and Figure 2, respectively. The flow of the game is as follows: the EGO vehicle chooses the strategy first and, then, the FV reacts to it, each trying to maximize the outcome of the interaction.

The outcome or total payoff can be defined as a set of payoff functions such as the payoff for time consumption, safety, and position. Each payoff depends on different parameters, including original position of the vehicles, gap size with respect to LEAD, driving style, cooperation degree, and urgency. The result can be predicted by defining the outcome as follows (see Figure 2): $P_{11}>P_{21}>P_{12}>P_{22}$ and $Q_{11}>Q_{21}>Q_{12}>Q_{22}$.

The FV will always choose the strategy denoted by B1 because, in this case, its payoff is the biggest. The EGO will always choose the strategy denoted by A1 strategy because $P_{11}>P_{12}$. Therefore, the players will always select the strategies A1 or B1 in order to obtain the $P_{11}$ and $Q_{11}$ payoffs.

Thus, by formalizing the payoffs in the form of a mathematical equation, it is possible to model the decision-making process of drivers and to predict the result of the game. In this paper, we propose a decision-making model that adopts the same approach. However, instead of the strategies A1, A2, B1, and B2, the strategy set of our proposed model is defined as acceleration ranges, as proposed in [13,15,16].

The acceleration range is defined as [0,$F(a_{LEAD})$]. The lowest acceleration cannot be a negative value, since our game starts when EGO stops at the adjacent lane between FV and LEAD and the players can select the strategy (acceleration) only once. The upper limit is constrained by the acceleration and position of LEAD, as it affects the possible acceleration strategies and payoff criteria of both EGO and FV. It is important to point out that LEAD is not a player of the game because it does not interact with FV and EGO. The proposed game including the set of strategies and the outcome values in its normal-form representation is depicted in Table 2.

### 3.2. The Payoff and Penalty Functions

The FV’s outcome is declared as the product of the penalty and the sum of the payoff functions, such as FV’s safety and space (namely, the desire to keep the current gap with the LEAD). The EGO’s outcome is the product of its safety payoff and the penalty. The difference between the EGO’s and FV’S safety payoffs is that the EGO’s safety payoff is based on the LEAD and FV vehicles while the FV’s safety payoff is based on the EGO vehicle.

The penalty function is implemented in order to include the impact of a driver’s driving performance into the decision-making model. It uses the average of the acceleration and the speed driving patterns to determine the driving performance in the other intersections previous to the last one, and it is then able to penalize drivers that deviate from these values. We based our model on the work in [16]. We adapted it by redesigning the initial payoff’s functions for suitability to our scenario, as described in detail through the functions in the following subsections:

#### 3.2.1. Safety Payoff Function of FV

The safety payoff function of FV, $U_{safety}^{FV}$ is defined by Equation (1) as the difference between the original safety function in time t0, $SF_{t0}^{FV}$, and the safety function in time ti, $SF_{ti}^{FV}$:

(1) $$U_{safety}^{FV}=SF_{t0}^{FV}-SF_{ti}^{FV}$$

The FV’s safety function $SF^{FV}\left(t\right)$ depends on the gap between the EGO and the FV and is denoted by Equation (2). In order to convert the function to the range [−1; 1], 0.5 is subtracted from the cumulative distribution function and then the result is multiplied by 2.

(2) $$SF^{FV}\left(t\right)=2\left(F_{cdf}\left(G_{EGO,FV}\right)-0.5\right))$$

where $F_{cdf}$ (see Equation (3)) is a cumulative normal distribution function, in which the value of variable x has been normalized, and the mean and standard deviation values adjusted to create the resulting function G(t).

**Figure 3:**
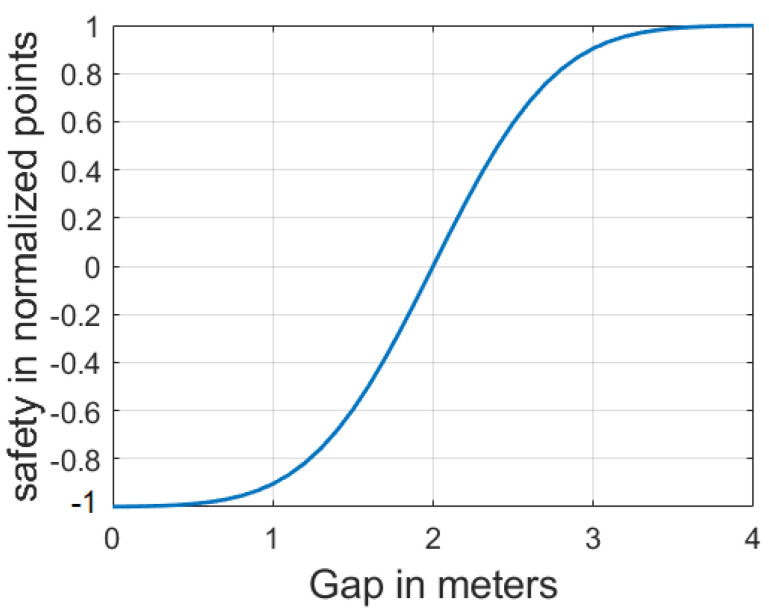
FV’s safety function represented by safety in the form of scored points and the headway or gap between vehicles in meters.

As illustrated in Figure 3 the mean value μ and standard deviation σ were adapted such that SF(t) equals −1 if $G_{v1,v2}\left(t\right)$ is less or equal zero and SF(t) equals 1 if $G_{v1,v2}\left(t\right)$ is greater than the defined 4 maximum gap in meters.

(3) $$F_{cdf}=\frac{1}{\left(\sigma \sqrt{2\pi }\right)}\exp(-\frac{{x-\mu )}^{2}}{2\sigma^{2}})$$

where: σ=0.6, μ=2, x = $G_{v1,v2}\left(t\right)$.

As denoted in Equation (4), the $G_{v1,v2}\left(t\right)$ function returns the distance between two vehicles at time t. In order to obtain the distance, it is necessary to subtract the mean length of the two vehicles from the difference of their longitudinal positions. x(t) is defined as the center of the vehicle.

(4) $$G_{v1,v2}\left(t\right)=x{\left(t\right)}_{v1}-x{\left(t\right)}_{v2}-\frac{l_{v1}+l_{v2}}{2}$$

where $x{\left(t\right)}_{vx}$ denotes the longitudinal positions and $l_{vx}$ denotes the length of the vehicles.

The longitudinal position x(t) is defined based on the uniform acceleration in Equation (5), where the value of variable *a* depends on the selected strategy.

(5) $$x\left(t\right)=x_{0}-V_{0}+\frac{at^{2}}{2}$$

#### 3.2.2. Space Payoff Function of the FV

Similar to safety payoff, space payoff for FV is defined as the difference between the values of space function SpF(t) in time t0 and time ti (Equation (6)).

(6) $$U_{space}^{FV}=SpF_{t0}^{FV}-SpF_{ti}^{FV}$$

**Figure 4:**
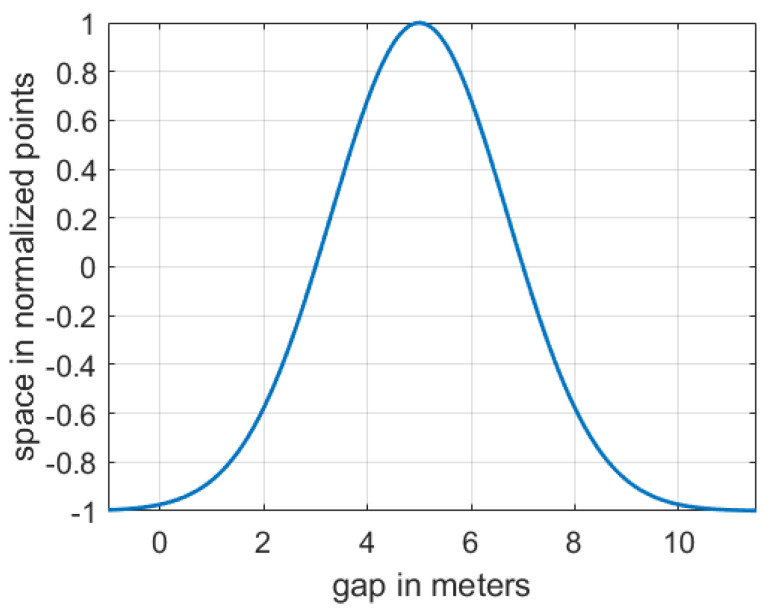
FV’s space function depending on the cooperation level regarding willingness to let EGO merge (scored points) and the headway or gap between FV and LEAD in meters.

The normalized probability density function denoted by Equation (7) is utilized to obtain the FV’s space payoff function. Similar to the FV’s safety payoff function, the *x* parameter was adapted in order to satisfy function $G_{v1,v2}\left(t\right)$, as denoted in Equation (4). Furthermore, σ and μ are adjusted in a way that SpF(t) equals 1 when the gap between FV and LEAD equals 5 and SpF(t) equals −1 when the gap is bigger than 10 or less than 0, as illustrated in Figure 4.

(7) $$SpF^{FV}\left(t\right)=2\left(\exp(-0.5\times \frac{\left(x-\mu \right)}{\sigma }\right)-0.5)$$

where: $\sigma =\frac{5}{3}$, μ=5, x = $G_{LEAD,FV}\left(t\right)$.

#### 3.2.3. Safety Payoff Function of EGO

The EGO’s safety payoff function is similar to the FV’s safety payoff function, as denoted by Equation (8). The only difference is that the EGO’s safety payoff function depends on both FV and LEAD.

(8) $$U_{safety}^{EGO}=SF_{t0}^{EGO}-SF_{ti}^{EGO}$$

The EGO’s safety payoff function $SF^{EGO}\left(t\right)$ is based on Equation (3) and is defined as the product of two cumulative distribution functions (Equation (9)). The first cumulative distribution function is a function of $G_{EGO,FV}\left(t\right)$, and the second one is a function of $G_{LEAD,EGO}\left(t\right)$. To illustrate the relationship between the EGO’s safety payoff function and the vehicle’s position, we present an example in which we use FV’s longitudinal position as the origin (0 m) and we set the length of all vehicles to 5 m.

**Figure 5:**
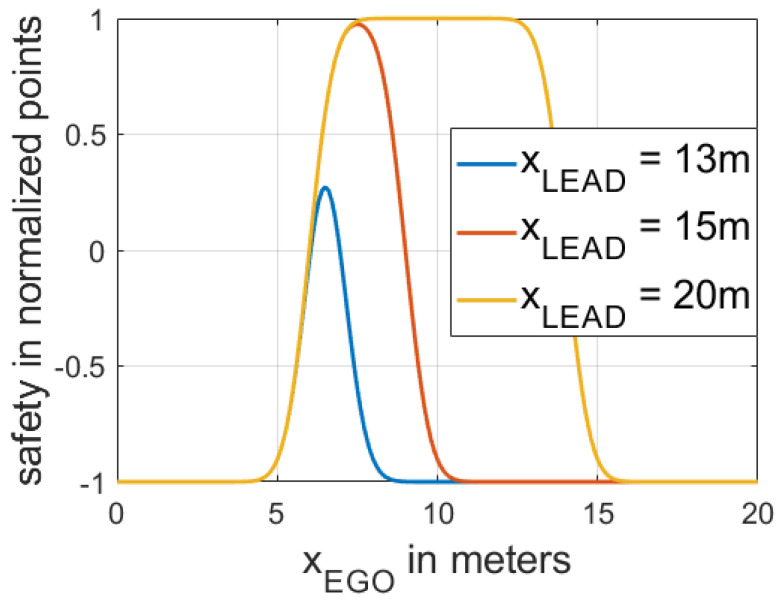
Dependencies between the EGO’s safety payoff function and the position of EGO ($x_{EGO}$). Note that $x_{LEAD}$ is the longitudinal position of LEAD, which determines the distance between FV and LEAD.

At the same time, three different LEAD cars’ longitudinal positions are chosen, which are used to demonstrate different situations in which the distance between LEAD and FV changes. Figure 5 shows the dependencies between the EGO’s safety function and the position of the EGO vehicle.

(9) $$\begin{array}{c} SF^{EGO}=2(F_{cdf}\left(G_{EGO,FV}\left(t\right)\right)\times \\ F_{cdf}\left(G_{LEAD,EGO}\left(t\right)\right)-0.5) \end{array}$$

#### 3.2.4. Penalty Function of the Vehicles

The original penalty function described in [16] was directly implemented to create our model, as it suited our considered scenario. We describe the process in this section. The penalty function is defined to consider the driving patterns of the vehicles that interact with EGO in the decision-making model. The function takes as input the FV acceleration and speed values acquired before stopping at the intersection. It calculates the outcome based on the deviation between these values and the selected FV and EGO parameter values for the decision-making process. The penalty function consists of the speed penalty $P_{V}$ and the acceleration penalty $P_{a}$, as shown in Equation (10).

(10) $$U_{penalty}=\exp\left(-\left(P_{V}+P_{a}\right)\right)$$

The speed and the acceleration penalty functions are obtained as the squared deviation of the acquired values before the stop at the final intersection and the selected values applied as a result of the decision to allow EGO to merge in front, as denoted by Equations (11) and (12).

The ws and wa coefficients are used to increase the impact of these functions on the total penalty.

(11) $$P_{V}=\frac{{\left(V_{0}+at-V_{a}\right)}^{2}}{ws}$$

where ws is the speed penalty coefficient and $V_{a}$ is the acquired speed prior to stopping at the last intersection.

**Figure 6:**
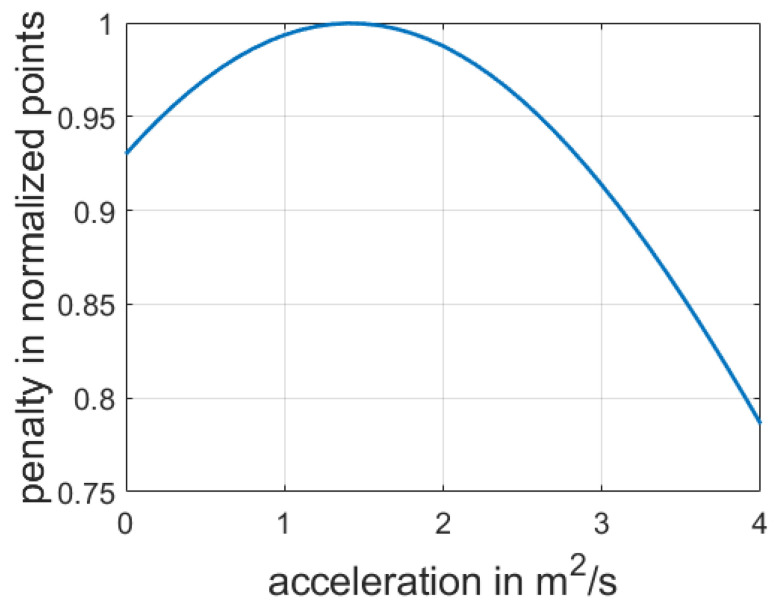
Penalty function, with $a_{a}=1.5\;{m/s}^{2}$, V=0m/s, t=3s, $V_{a}=5\;m/s$, wa=500, and wd=500.

Figure 6 displays the dependency of the penalty function and a selected acceleration strategy. The greater the deviation from the desired option, the less the payoff value will be.

(12) $$P_{a}=t^{2}\frac{{\left(a-a_{a}\right)}^{2}}{wa}$$

where wa is the coefficient of acceleration penalty and $a_{a}$ is the acquired acceleration prior to stopping at the last intersection.

#### 3.2.5. The Outcome of the Vehicles

The outcome for both players is illustrated in Equations (13) and (14).

(13) $$U_{EGO}=U_{safety}^{EGO}\times U_{penalty}$$

The outcome of FV is based on the mean value of its safety and space payoff functions. In the proposed model, we assume that the FV’s space payoff has the same impact on the driver as the safety payoff. Therefore, in Equation (14), we use the mean value between these two payoff functions.

(14) $$U_{FV}=\left(\frac{U_{safety}^{FV}+U_{penalty}^{FV}}{2}\right)U_{penalty}$$

## 4. Data Acquisition and Use Case Definition for Model Validation

In order to validate the proposed decision-making model, we designed a simulation environment that replicates the scenario described in Section 1. To this end, we used the simulator for cooperative advanced driver assistance systems (ADAS) and automated vehicles, 3DCoAutoSim, which was developed to test intelligent transportation systems (ITS)-related applications and connects the game engine Unity 3D [22] with Simulation of Urban MObility (SUMO) [23] and ROS [24,25,26,27,28,29,30]. Details of the simulation framework are presented in the following subsection.

**Figure 7:**
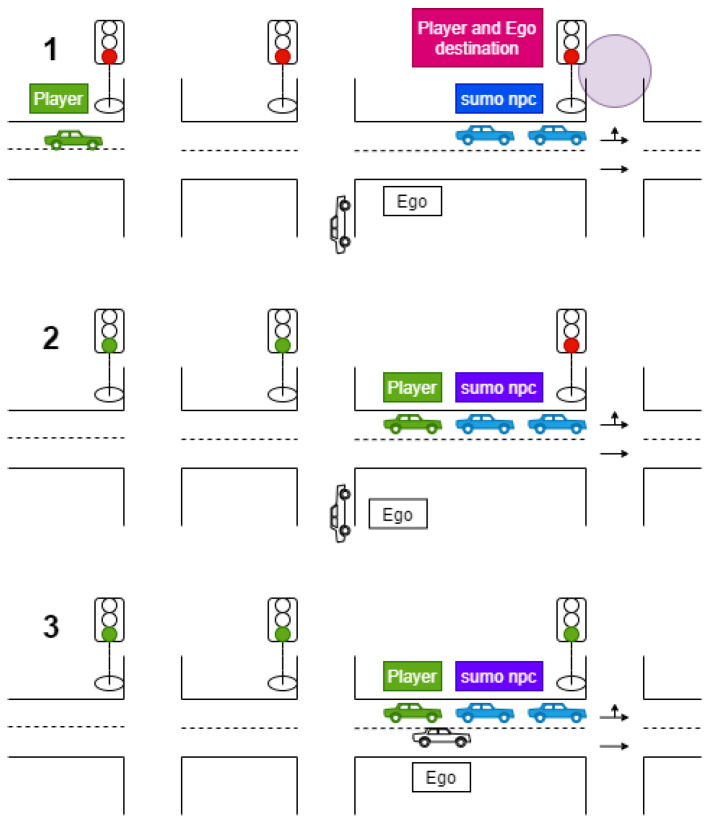
Model validation scenario: 1. The player’s vehicle (FV) approaches the target intersection. 2. The player’s vehicle enters the queue to wait for the traffic light to turn green. EGO is activated to move to the left lane; 3. EGO vehicle is located at the position defined in the proposed decision-making model.

The participants manually controlled the player vehicle (Player, denoted until now with FV). They were asked to drive from the origin to destination within a given time interval while following traffic rules. In order to acquire the necessary data regarding driving patterns, the main route included several intersections. At the last intersection, the traffic lights were controlled by a specific program so that they remained red until Player and EGO were both located at the positions defined in the proposed decision-making model (Figure 7(1)).

When Player stopped at the final intersection and entered the queue of traffic waiting for the traffic lights to turn green, an event was triggered for EGO to drive to the left lane in which Player was located (Figure 7(2)).

When EGO stopped and activated the left turn signal to perform the lane change, a further event was triggered to apply the proposed decision-making model (Figure 7(3)).

**Figure 8:**
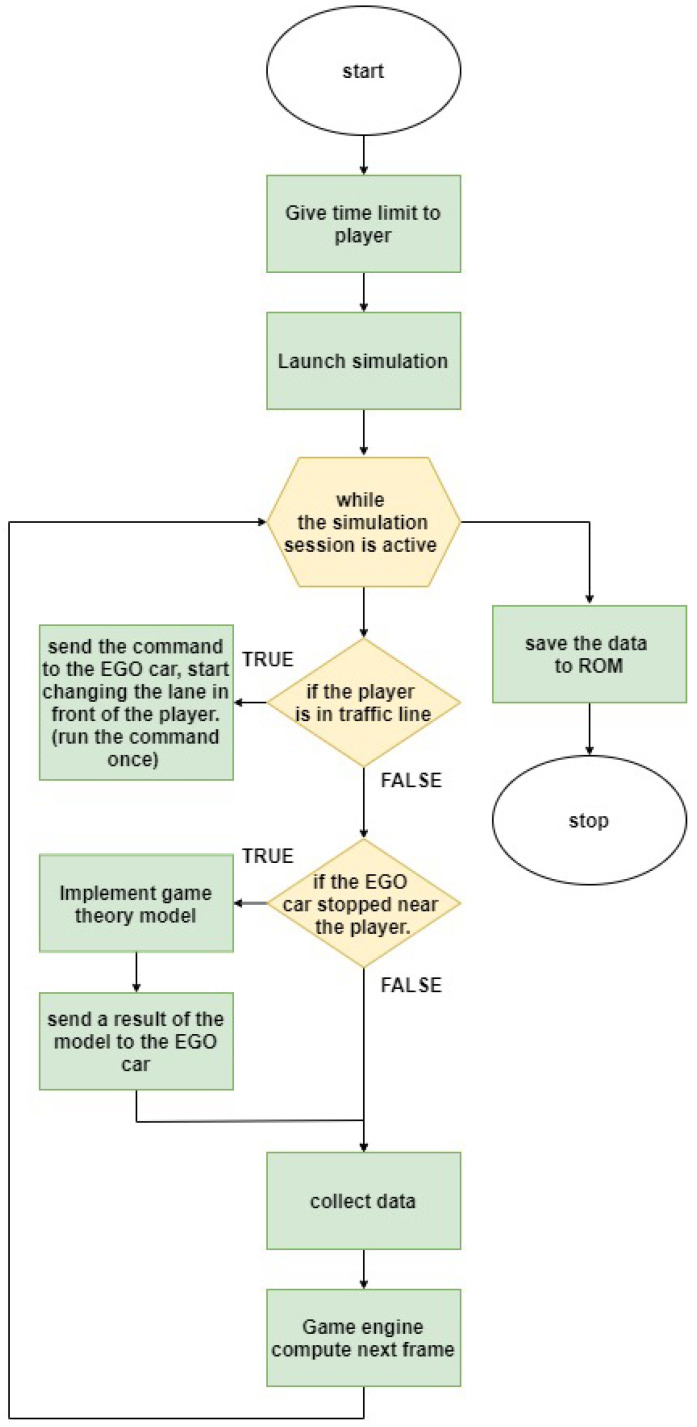
Flow chart describing the validation process.

The last step of the simulation included the interaction between Player and EGO, during which the participants in the driving test needed to decide whether they accepted or rejected EGO’s request to change lanes and to merge. Figure 8 illustrates the validation process flow.

### 4.1. 3DCoAutoSim Description

As previously mentioned, to perform the pertinent driving experiments to validate the proposed model, we relied on the 3DCoAutoSim framework, which is a driver-centric simulator in which the player controls a vehicle that drives through created or imported scenarios. The simulator is used in order to test different use cases or applications regarding the feasibility of automation and communication features in vehicles. It was developed using the cross platform game engine Unity 3D for developing 3D games with simulated physics [22].

**Figure 9:**
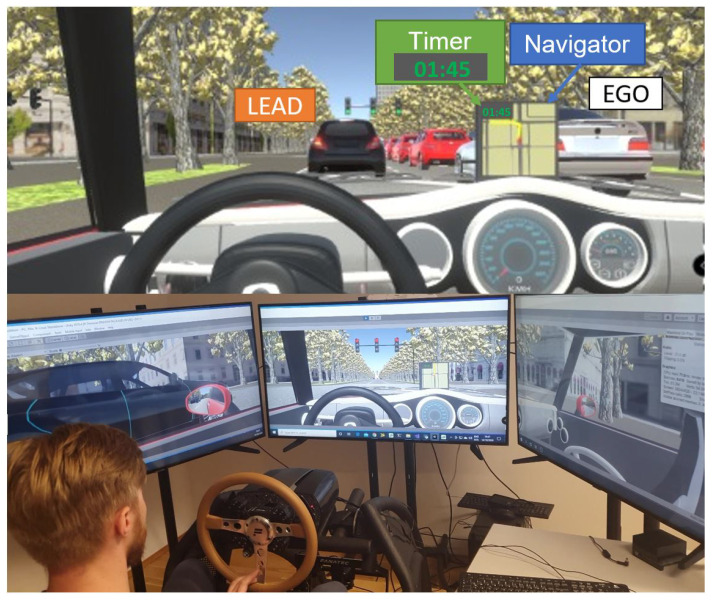
The 3DCoAutoSim simulation platform during a driving test and a screenshot of the scenario setup and in-vehicle systems.

The Player vehicle is controlled using the CSL Elite Wheel Base and pedals from Fanatec, which communicates with Unity3D through an external intrinsic input module of the game engine, as illustrated in Figure 9.

To simulate and visualize traffic, the modular components of 3DCoAutoSim are linked with SUMO to communicate through the traffic control interface (TraCI), which allows external applications to access a running road traffic simulation and to obtain the values of the simulated objects to manipulate their behavior online. Unity uses a C# library of TraCI to access the SUMO parameters and to consequently generate traffic.

### 4.2. SUMO Description

**Figure 10:**
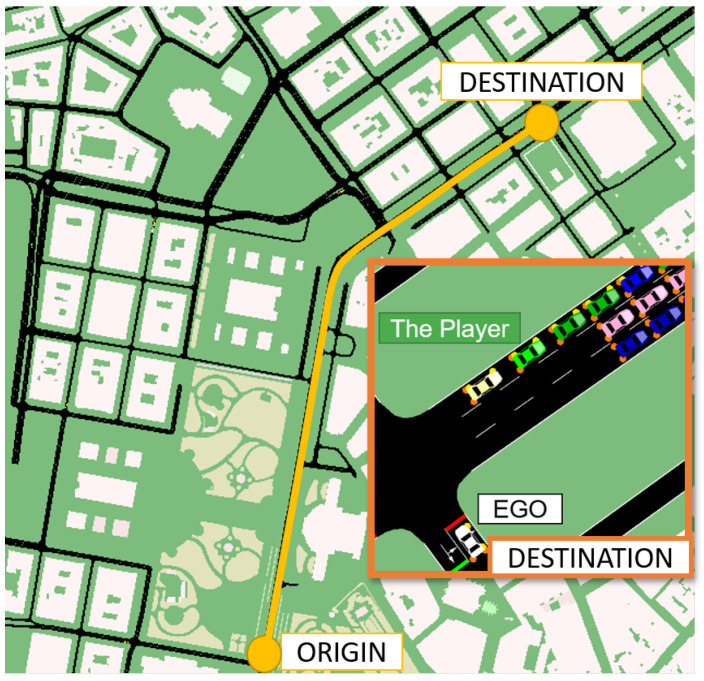
Trip origin and destination on the generated Simulation of Urban MObility (SUMO) road network to perform the model validation with a section of the generated SUMO simulation with different vehicle types at the destination.

**Figure 11:**
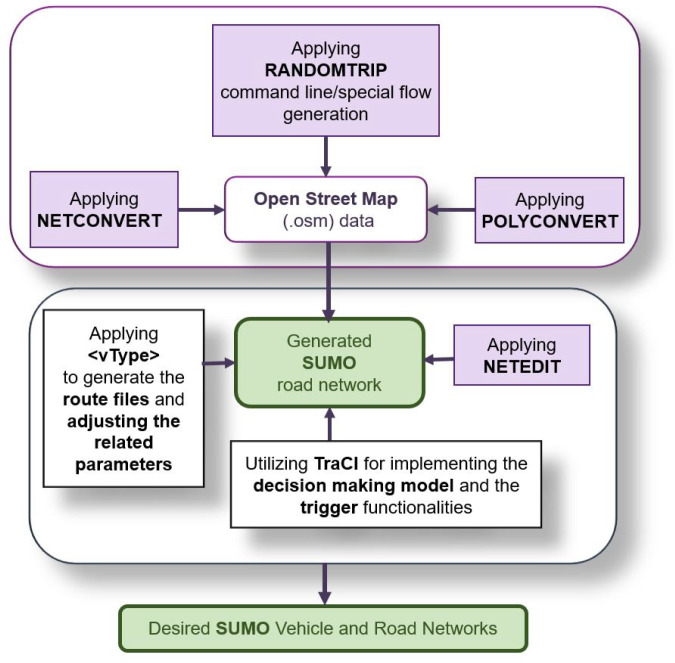
SUMO simulation steps (adapted from [35]).

In the model validation scenario, SUMO controlled all the traffic lights and vehicles in the scenario except the Player vehicle. In order to generate the SUMO road and traffic network simulation, a section of the city of Vienna was imported from OpenStreetMap (OSM) [31] into SUMO. The NETCONVERT [32] and POLYCONVERT [33] commands were then applied to the OSM data to generate the road network as illustrated in Figure 10. To generate the traffic demand, a set of random trips [34] with different vehicle types (vType elements) were also added to the network (Figure 10). The process to generate and visualize the traffic in SUMO is presented in Figure 11.

In addition, we adopted TraCI to study the performance of the proposed decision-making model and the trigger models.

In order to trigger the intended behavior of the EGO vehicle in the congested traffic intersection, we developed a procedure with different behavioral levels, as illustrated in Algorithm 1. If the Player parameter RoadID matches the ID of the last intersection while waiting for the traffic light to turn green, a trigger for EGO is activated for it to merge in front of the Player on the left lane (Figure 7).
**Algorithm 1** Trigger for Activating the EGO Vehicle**input**: Player class. pv; RoadID of the last intersection, rID; **output**: event *t*; 
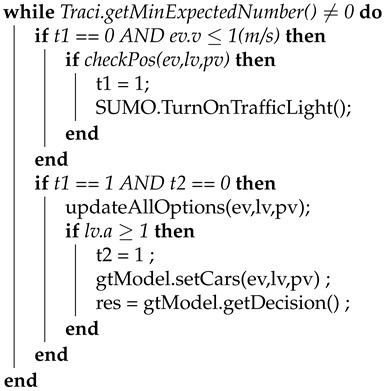


Algorithm 2 shows the procedure developed to investigate the performance of the proposed decision-making model. The EGO vehicle is first verified in the algorithm. If the EGO is stopped and its left turn signal is activated to perform a lane change, an event to apply the proposed decision-making model is triggered for the Player. By applying the decision-making model, the Player decides whether to accept or reject the lane-change request from EGO.

### 4.3. Experimental Setup

We describe in this section the experimental setup.

Sample: eight persons took part in the model validation process through several driving tests. The group included 6 males and 2 females with driving experience ranging from 0.5 year to 31 years and an average age of 31 years. A total of 16 trips from 2 rounds, one without a time limit and one with a time limit, were conducted, from which data were collected.
**Algorithm 2** Proposed game theory-based decision-making model.**input**: Player vehicle class, pv; LEAD vehicle class, lv; EGO vehicle class, ev; **output**: Bool res; t1 = 0, t2 = 0; 
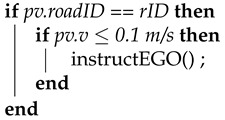


Task definitions: participants in the experiment were required to perform the following tasks:

1. Drive for 5 min to become familiarized with the simulation platform. No data were collected.
2. Drive from the origin to destination, as described in the first paragraphs of Section 4.
3. Drive from the origin to destination, as described but with a time limitation. This task aimed to add pressure and urgency and to motivate the participant to reject a lane-change request from the EGO side.

Generated scenario: the generated scenario to validate the proposed model depicted a section of the city of Vienna center (Vienna Ring). The structure and the position of the buildings were imported from the Vienna Magistrate’s database [36]. The free and open source 3DBlender software [37] was used to convert and edit the buildings to the Unity format. We additionally equipped the participant vehicle (Player, FV) with a routing navigation system (Navigator) to guide them through the route. A timer completed the setup of the vehicle to notify drivers of the time elapsed (see Figure 9).

## 5. Results

### 5.1. Game Solution Description

By relying on the outcome of the previously defined method, we describe in this section the solution for the proposed approach.

Equation (15) shows a bi-level optimization problem, in which the optimal EGO’s strategy $a_{ego}^{*}$ depends on the selected strategy by $a_{FV}^{*}$.

In our proposed decision-making model, we assumed that EGO is aware of the driving behavior of FV prior to stopping at the intersection. Therefore, the EGO can estimate and predict which would be the strategy that FV would pursue and can make a decision accordingly such that EGO can reach its maximum game outcome.

(15) $$a_{EGO}^{*}=\underset{a_{ego}}{\arg\;\max}\left(\underset{a_{FV}^{*}\in S_{FV}^{*}}{U_{EGO}\left(a_{EGO},a_{FV}^{*}\right)}\right)$$

where $S_{FV}^{*}\subset S_{FV}$; meanwhile, with $\forall a_{FV}^{*}\in S_{FV}^{*}$, $\forall a_{FV}\notin S_{FV}^{*}$, and $\forall (a_{EGO},a_{FV})$, the inequality $U_{FV}\left(a_{EGO},a_{FV}^{*}\right)\geq U_{FV}\left(a_{EGO},a_{FV}\right)$ is satisfied.

**Figure 12:**
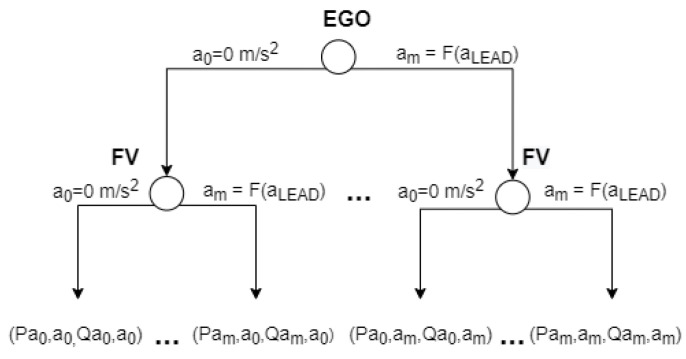
Dynamic noncooperative game description in its extensive-form representation. EGO and FV denote the players; $a_{0}$ and $a_{m}$ denote their respective sets of strategies. Pxy and Qxy denote the outcome of the game, where x is the FV’s selected strategy and y is the strategy selected by EGO.

In order to simplify the process of solving the task, we used discrete acceleration values with a step equal to 0.1 m/s${}^{2}$. Therefore, the game can be solved by first obtaining the FV’s set of optimal strategies $S_{FV}^{*}$ based on any of EGO’s strategies $a_{EGO}$ and then by using the obtained set $S_{FV}^{*}$ to find EGO’s optimal strategy $a_{EGO}^{*}$. The extensive-form of the proposed game is illustrated in Figure 12.

The result of the game can therefore be implemented in the proposed decision-making model in the following way.

If $a_{EGO}^{*}>a_{FV}^{*}$, the lane change can be performed; otherwise, a collision might occur.

### 5.2. Driving Experiment Prediction Results

Regarding the results from the driving experiment performed in the simulator to validate the decision-making model proposed in this paper, Table 3 shows the input values for the proposed model collected during the experiment. In lines 1 and 12, the model prediction values did not match the action of the drivers. In 11 of the 16 cases in which FV needed to make a decision to accept or reject the lane-change request by the EGO, FV rejected the request and EGO did not perform the lane-change maneuver. In the other 5 cases for requests, FV enabled the lane change. The proposed decision-making model correctly predicted all 5 outcomes and 9 out of 11 outcomes in the cases in which FV rejected the request from EGO. Table 4 shows these results. Delay from the side of FV in resuming driving after the traffic light turned green (see *a* and speed *v* values close to zero in tests 1 and 12 in Table 3) affected the safety payoff function, in this case, the model making the decision to perform the lane-change maneuver. Equations (16) and (17) were used to estimate the acquired speed and acceleration values of Player prior to stopping at the last intersection.

(16) $$a_{a}=mean\left(a>0.01\right)$$

(17) $$v_{a}=mean\left(v>0.3\right)$$

The presented data in Table 3 shows that, in the considered scenario, the gap size had a significant impact on the driver’s final decision: the bigger the gap between the FV and the LEAD, the higher the probability for the driver to accept the lane-change request from the EGO vehicle. At the same time, some exceptions could also been observed in lines 1 and 12. They were related to the time it took the drivers to resume driving after the traffic light turned green. The speed and acceleration were also determining factors on the final decision in the considered scenario. The effect can be noticed when comparing test 4 with test 13. In test 4, the gap is bigger (5.31 m) compared with test 13 (3.79 m), but despite this fact, the driver in test 4 rejected the lane-change request from the EGO car while the driver in test 13 accepted the request. We argue that this behavior was due to the fact that the acceleration and speed values at an instant of time right before being used to calculate the prediction in test 4 were higher than in test 13 even if the acquired speed and acceleration values of Player prior to stopping at the last intersection were similar.

Summarizing all the results presented in the Table 3 and Table 4, we can conclude that the proposed decision-making model accurately predicts the drivers decisions regarding whether they allow the autonomous vehicle to drive in front of them.

## 6. Conclusions and Future Work

We presented in this work a dynamic noncooperative decision-making model based on a two-player nonzero-sum game theory. The goal was to provide autonomous vehicles with capabilities for analyzing human decision-making processes and for acting accordingly. The ultimate goal was to facilitate the coexistence of manual and autonomous vehicles in a mixed environment to augment road safety.

The results from validation of the proposed game theory-based decision-making model are promising, as the prediction rate was 100% in all of the cases in which the participants allowed the lane change and 83.3% in the other cases, affected by the driver’s reaction time to the traffic light color change from red to green. The driver’s final decision to accept a lane-change request from the EGO vehicle was influenced by the gap size between vehicles and by the speed and acceleration of the Player vehicle.

We conclude that our approach can be used as a tactical-level algorithm for making lane-changing decisions in autonomous vehicles in congested urban intersections. The reliability and prediction accuracy can be increased by evaluating the decision-making process during the entire lane-change action with a fixed “n” millisecond time step. Future work will include this idea and will extend the current approach to a game theory-based model with incomplete information using quantum response equilibrium. This will make it possible to address scenarios in which the EGO vehicle is not capable of obtaining all of the required information from the vehicles in the target lane. In addition, the driver’s reaction time will be considered in the payoff functions and the proposed model will be generalized such that it can be applied to both not crowded and crowded urban scenarios.

## Figures and Tables

**Table 1:** Dynamic noncooperative game description in its normal-form representation. EGO and FV denote the players; A1 and A2, and B1 and B2 denote their respective sets of strategies. Pxy and Qxy denote the outcome of the game, where x is the FV’s selected strategy and y is the strategy selected by EGO.

Action	EGO Vehicle	
	*A*1 (Change Lane)	*A*2 (Do Not Change Lane)
The FV vehicle		
*B*1 (Accept)	$\left(P_{11},Q_{11}\right)$	$\left(P_{12},Q_{12}\right)$
*B*2 (Decline)	$\left(P_{21},Q_{21}\right)$	$\left(P_{22},Q_{22}\right)$

**Table 2:** Game description in its normal-form representation. EGO and FV denote the players. $a_{EGO}$ and $a_{FV}$ denote the sets of strategies for the EGO and FV, respectively. $P_{a_{FV},a_{EGO}}$ and $Q_{a_{FV},a_{EGO}}$ denote the outcome values of the game with respect to the players.

Action	EGO Vehicle	
	$0\leq a_{\mathrm{EGO}}\leq F\left(\mathrm{LEAD}\right)$
FV	
$0\leq a_{FV}\leq F\left(LEAD\right)$	$\left(P_{a_{FV},a_{EGO}},Q_{a_{FV},a_{EGO}}\right)$

**Table 3:** Input values regarding the driving performance data used to predict cooperation for an EGO merging maneuver. $a_{a}$ and $v_{a}$ denote the acquired speed and acceleration values of Player prior to stopping at the last intersection, *a* and *v* are the acceleration and speed values at an instant of time right before being used to calculate the prediction, gap is the existent gap between Player and LEAD, action denotes the player’s decision, and predicted is the model prediction value.

test	$a_{a}$ ${m/s}^{2}$	$v_{a}$ m/s	*a* m/s${}^{2}$	*v* m/s	gap m	action	predicted
1	0.84	8.61	0.01	0.07	7.07	reject	accept
2	1.48	12.01	0.04	0.95	3.50	reject	reject
3	0.80	6.84	0.00	0.00	7.72	accept	accept
4	1.17	8.98	0.02	0.56	5.31	reject	reject
5	1.34	9.65	0.00	0.00	7.07	accept	accept
6	1.42	11.53	0.02	0.27	2.92	reject	reject
7	1.22	10.62	0.03	0.34	1.36	reject	reject
8	2.11	12.89	0.00	0.21	3.11	reject	reject
9	1.08	10.33	0.02	0.17	5.17	accept	accept
10	1.44	12.03	0.03	0.59	3.25	reject	reject
11	1.03	7.79	0.2	0.27	4.76	reject	reject
12	1.03	8.77	0.00	0.01	4.41	reject	accept
13	1.16	8.41	0.00	0.00	3.79	accept	accept
14	1.80	11.29	0.03	0.48	1.93	reject	reject
15	1.24	10.81	0.00	0.00	7.53	accept	accept
16	2.08	14.28	0.02	1.14	5.21	reject	reject

**Table 4:** Confusion matrix of the validation test.

n = 16	Predicted: NO	Predicted: YES	
Actual: NO	9	2	11
Actual: YES	0	5	2
	9	7	

## Data Availability

Not applicable.
